# Supplementary material for: Mitorubin, berberrubine-based compounds that improve mitochondrial function, exhibit cardioprotective effects against age-related cardiac dysfunction
Source: NPJ Aging. 2026 Mar 20;12(1):56. doi: 10.1038/s41514-026-00366-w (PMC13091788; doi:10.1038/s41514-026-00366-w)
Supplement: Supplementary file 1 — Supplementary Information [file 41514_2026_366_MOESM1_ESM.pdf]

Supplementary Information

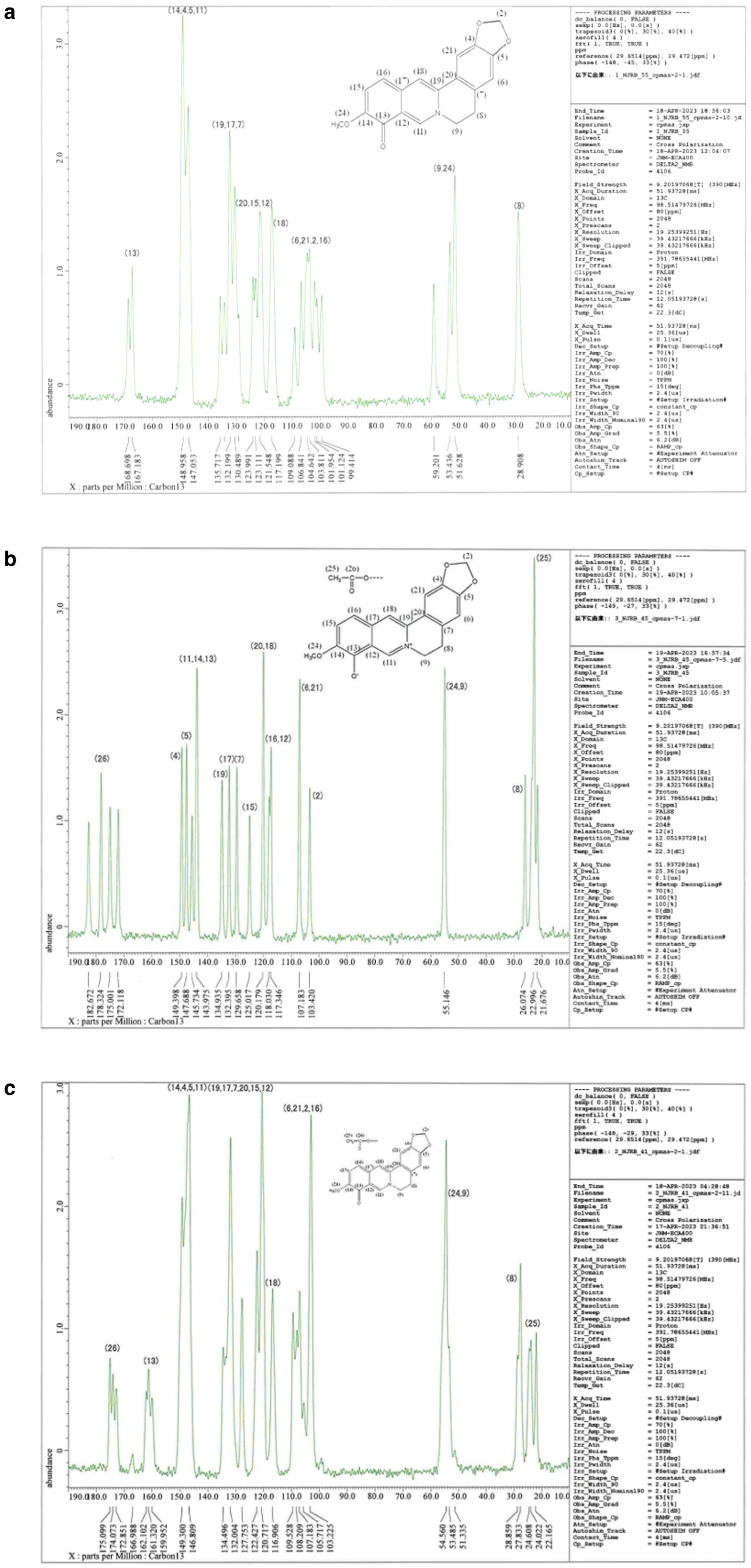

Supplementary Figure 1.
Structural analysis of quinoid-type berberrubine, berberrubine acetic acid adduct, and berberrubine acetic acid salt.

Solid-state ^{13}C-NMR spectra of the berberine-derived compounds were obtained using the CPMAS method. The spectra are presented from top to bottom: **(a)** reddish-black solid (quinoid-type berberrubine), **(b)** reddish-brown solid (berberrubine acetic acid salt), and **(c)** yellow solid (berberrubine acetic acid adduct).

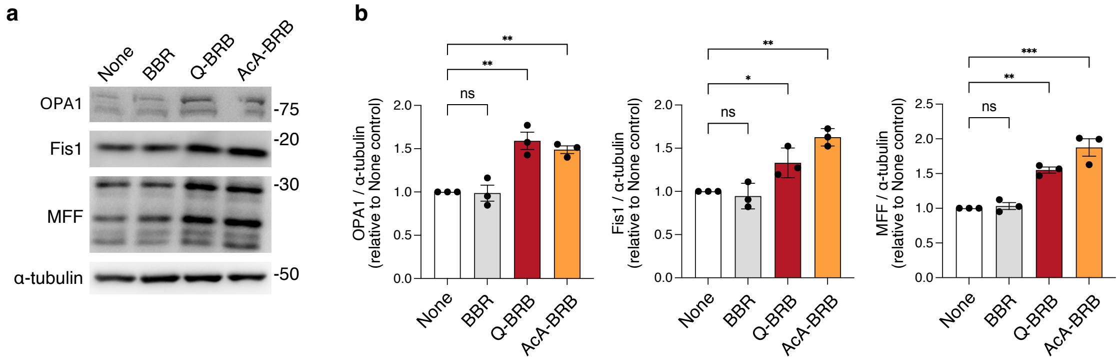

Supplementary Figure 2.
Upregulation of mitochondrial dynamics-related proteins by berberrubine in C2C12 myoblasts.

C2C12 cells were treated with quinoid-type berberrubine (Q-BRB) or the berberrubine acetic acid adduct (AcA-BRB) (10 μM) for 48 h, and protein expression levels of mitochondrial dynamics-related proteins (OPA1, Fis1, and MFF) were analyzed by western blot. Representative western blots are shown in **a**, and **b** shows the quantification of their signal intensities normalized to α-tubulin and expressed as values relative to the None group (n = 3 per group). The None group indicates cells cultured without any additives, including solvents.
Statistical significance was assessed using one-way ANOVA followed by Tukey's HSD test. *$p < 0.05$; **$p < 0.01$; ***$p < 0.001$; ns, not significant.

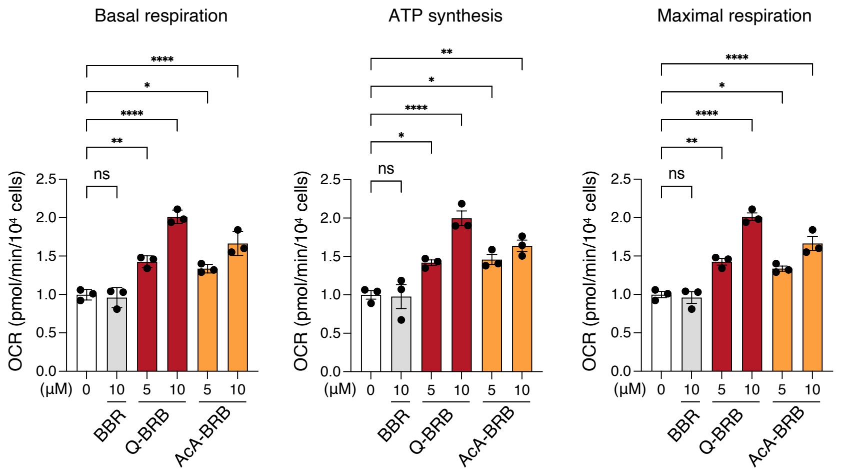

Supplementary Figure 3.
Enhanced mitochondrial oxygen consumption by berberrubine in H9c2 cardiomyocytes.

Rat cardiomyocyte H9c2 cells were treated with quinoid-type berberrubine (Q-BRB) or the berberrubine acetic acid adduct (AcA-BRB) (5 or 10 μM) for 48 h, and mitochondrial oxygen consumption rate (OCR) was measured (n = 3 per group). Basal respiration, ATP synthesis-linked respiration, and maximal respiration were calculated from the OCR data according to the method described in the Materials and Methods section. The None group indicates cells cultured without any additives, including solvents.
Statistical significance was assessed using one-way ANOVA followed by Tukey's HSD test. *$p < 0.05$; **$p < 0.01$; ****$p < 0.0001$; ns, not significant.

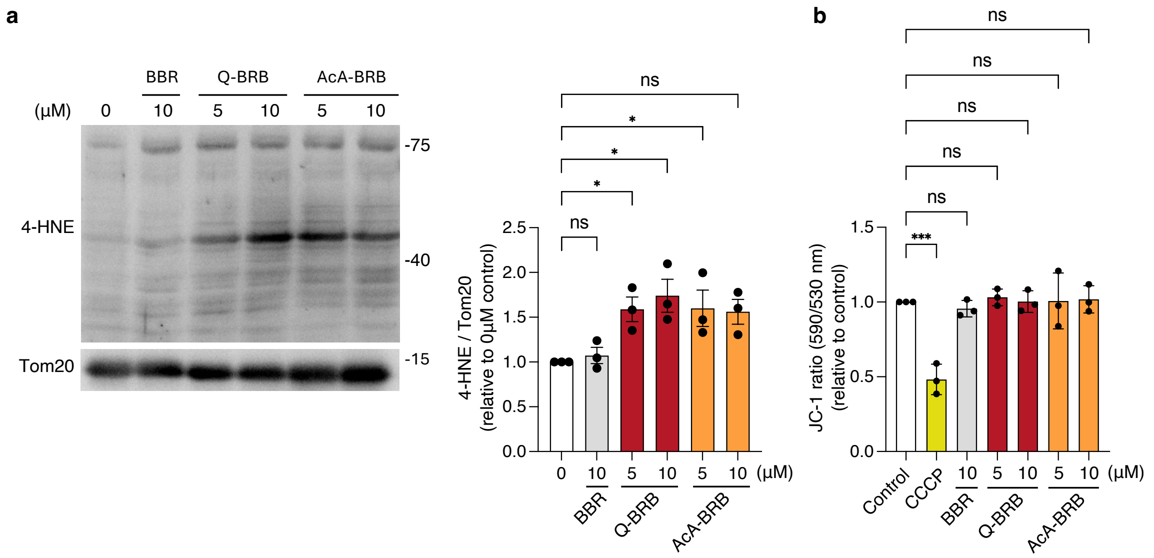

Supplementary Figure 4.

Assessment of mitochondrial ROS levels and membrane potential following berberrubine treatment.

(a) Mitochondrial ROS levels were evaluated by measuring 4-hydroxynonenal (4-HNE) in isolated mitochondrial fractions. C2C12 cells were treated with quinoid-type berberrubine (Q-BRB) or the berberrubine acetic acid adduct (AcA-BRB) (5 or 10 μM) for 24 h, and mitochondrial fractions were subjected to western blotting. Representative immunoblots of 4-HNE and Tom20 are shown on the left, and the quantification of 4-HNE/Tom20 ratios (expressed relative to the 0 μM control) is shown on the right (n = 3 per group).

(b) Mitochondrial membrane potential was assessed using the JC-1 dye. C2C12 cells were treated with Q-BRB or AcA-BRB (5 or 10 μM) for 48 h and stained with JC-1. The fluorescence ratio of red (590 nm) to green (530 nm) was quantified and expressed relative to the untreated control (n = 3 per group). CCCP (10 μM, 6 h) was used as a positive control for membrane depolarization.

Statistical significance was assessed using one-way ANOVA followed by Tukey's HSD test. *$p < 0.05$; **$p < 0.01$; ***$p < 0.001$; ns, not significant.

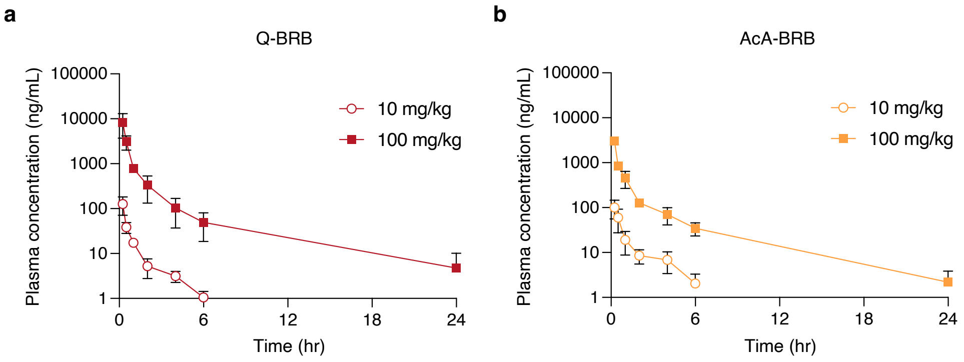

c

Compounds	Dose (mg/kg)	C_{max} (ng/mL)	T_{max} (hr)	$t_{1/2}$ (hr)	AUC_{∞} (ng/mL·hr)	CL_{tot}/F (mL/min/kg)	V_{dz}/F (L/kg)
Q-BRB	10	126	0.25	1.49	76.5	2180	281
	100	8342	0.25	4.98	5107	326	141
AcA-BRB	10	101	0.25	1.68	95.7	1742	253
	100	3055	0.25	4.31	2129	783	292

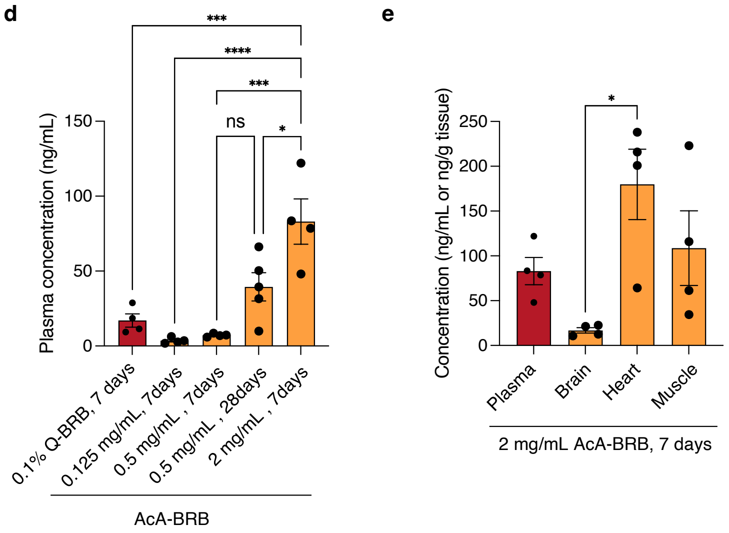

Supplementary Figure 5.
Pharmacokinetic evaluation of quinoid-type berberrubine and berberrubine acetic acid adduct in mice.

(a, b) Plasma concentration profiles of quinoid-type berberrubine (Q-BRB, **a**) and berberrubine acetic acid adduct (AcA-BRB, **b**) following a single oral gavage administration at low (10 mg/kg) and high (100 mg/kg) doses (n = 3 per group). Data are presented as mean ± standard deviation (SD).
(c) Pharmacokinetic parameters, including maximum plasma concentration (C_{max}), time to reach Cmax (T_{max}), elimination half-life ($t_{1/2}$), area under the plasma concentration-time curve (AUC_{∞}), apparent total clearance (CL_{tot}/F), and volume of distribution (V_{dz}/F), for Q-BRB and AcA-BRB at each dose.
(d) Plasma berberrubine concentrations in mice after 7-day administration of either 0.1% Q-BRB mixed in diet or AcA-BRB in drinking water at concentrations of 0.125, 0.5, and 2.0 mg/mL (n = 4 per group), including an additional 28-day group for the 0.5 mg/mL dose (n = 5).
(e) Plasma and tissue distribution of berberrubine in the brain, heart, and skeletal muscle of mice after 7-day administration of 2 mg/mL AcA-BRB in drinking water (n = 4 per group).
Statistical significance was determined using one-way ANOVA followed by Tukey's HSD test (**d**) and a two-sided unpaired Student's *t*-test (**e**). *$p < 0.05$; **$p < 0.01$; ***$p < 0.001$; ns, not significant.

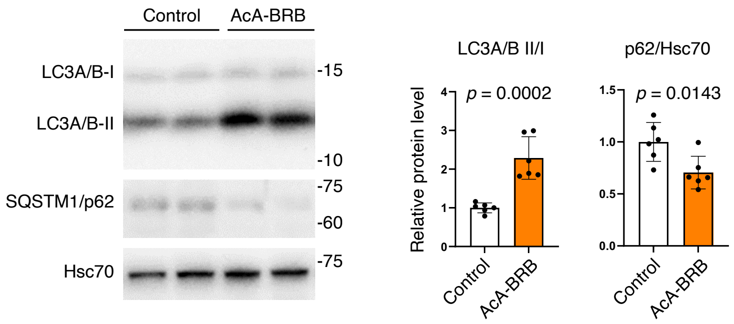

Supplementary Figure 6.
Evaluation of autophagy in heart tissues.

Representative western blots (left) and corresponding quantification (right) of heart tissues from the indicated mice, probed with antibodies against LC3A/B and SQSTM1/p62 (n = 6 per group). For LC3A/B, the LC3A/B II/I ratio was evaluated. Hsc70 served as a loading control. Values in control group were set to 1.
Statistical significance was determined using a two-sided unpaired Student's *t*-test.

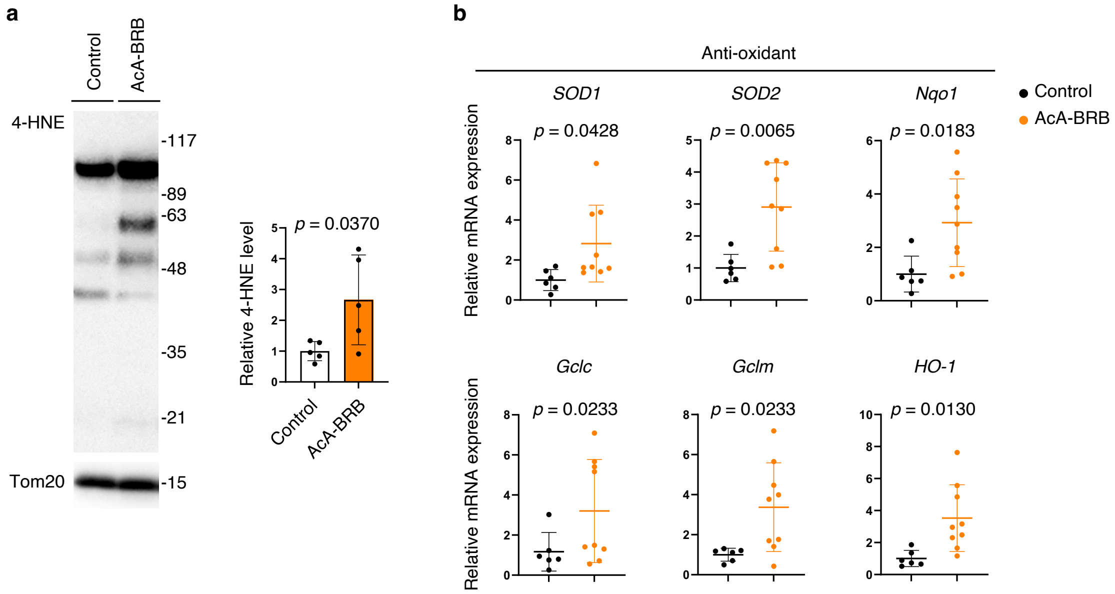

Supplementary Figure 7.
Assessment of ROS in isolated cardiac mitochondria and expression of anti-oxidant–related genes in heart tissues.

(a) Representative western blots (left) and corresponding quantification (right) of isolated cardiac mitochondria from the indicated mice, probed with an antibody against 4-hydroxy-2-nonenal (4-HNE) (n = 5 per group). Tom20 was used as a loading control. Values in the control group were normalized to 1.
(**b**) Relative expression of genes associated with anti-oxidant pathways in hearts of the indicated mice. Values in the control group were normalized to 1 (n = 6, control; n = 9, AcA-BRB).
Statistical significance was determined using a two-sided unpaired Student's *t*-test.

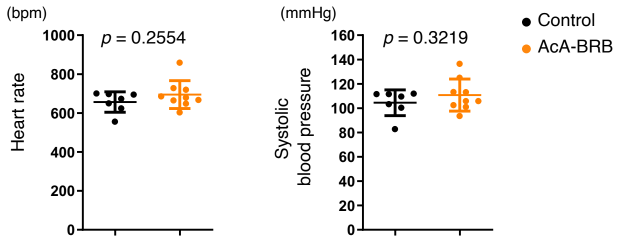

Supplementary Figure 8.
Heart rate and blood pressure in berberrubine-treated aged mice.

Heart rate (bpm, left) and systolic blood pressure (mmHg, right) in 24-month-old WT mice after 3 months of treatment with berberrubine or tap water (control) (n = 7, control; n = 9, AcA-BRB). Statistical significance was determined using a two-sided unpaired Student's *t*-test.

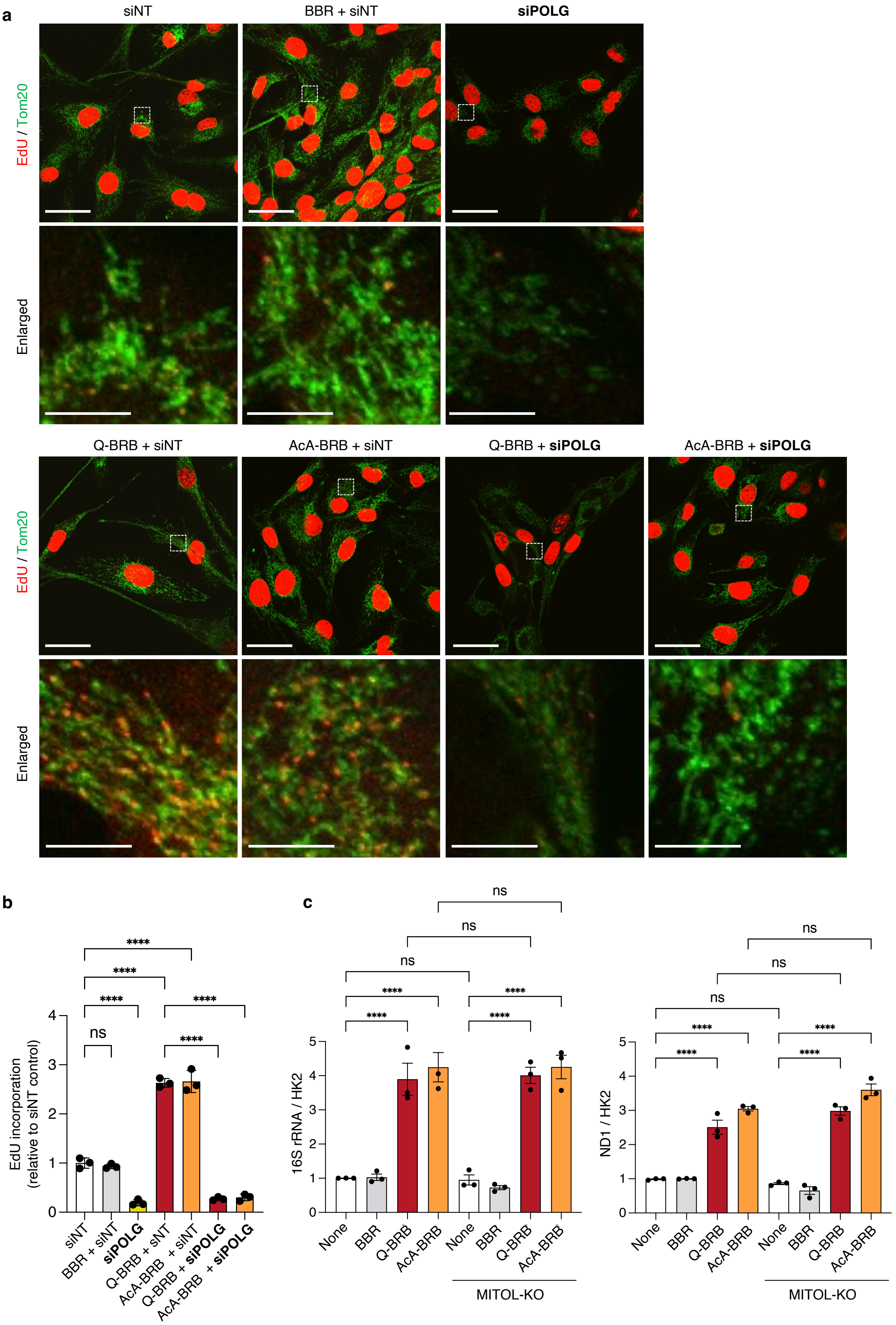
a
siNT
BBR + siNT
siPOLG
EdU / Tom20
Enlarged
Q-BRB + siNT
AcA-BRB + siNT
Q-BRB + siPOLG
AcA-BRB + siPOLG
b
EdU incorporation (relative to siNT control)
ns
****
siNT
BBR + siNT
siPOLG
Q-BRB + siNT
AcA-BRB + siNT
Q-BRB + siPOLG
AcA-BRB + siPOLG
c
16S rRNA / HK2
ND1 / HK2
ns
****
None
BBR
Q-BRB
AcA-BRB
MITOL-KO

Supplementary Figure 9.
Mechanistic analyses of berberrubine-induced increases in mitochondrial DNA content.

(a) Representative confocal images of EdU incorporation under the indicated conditions. C2C12 cells were transfected with non-targeting siRNA (siNT) or siPOLG and treated with berberine (BBR), quinoid-type berberrubine (Q-BRB), or the berberrubine acetic acid adduct (AcA-BRB) (10 µM) for 48 h. EdU labeling (red) marks newly synthesized DNA and is predominantly nuclear; mitochondrial DNA-associated EdU signals were defined as EdU-positive puncta colocalizing with the mitochondrial marker Tom20 (green). Enlarged views of the boxed regions highlight these colocalizing puncta. Scale bars: 50 µm (upper panels), 1 µm (lower panels).
(b) Quantification of mitochondrial EdU-positive puncta. EdU-positive puncta colocalizing with Tom20 were quantified from maximum-intensity projection images using a consistent threshold in Fiji. For each biological replicate (n = 3), ≥30 cells were randomly selected and analyzed. Data are presented as values relative to the siNT control group.
(c) Mitochondrial DNA content was quantified in wild-type and MITOL-knockout (MITOL-KO) C2C12 cells following treatment with Q-BRB or AcA-BRB (10 µM) for 48 h. Relative mtDNA levels were assessed by real-time PCR using the ratios of 16S rRNA/HK2 and ND1/HK2, and expressed as fold change normalized to the None group (n = 3 per group).
Statistical significance was assessed using one-way ANOVA followed by Tukey's HSD test. ****p < 0.0001; ns, not significant.

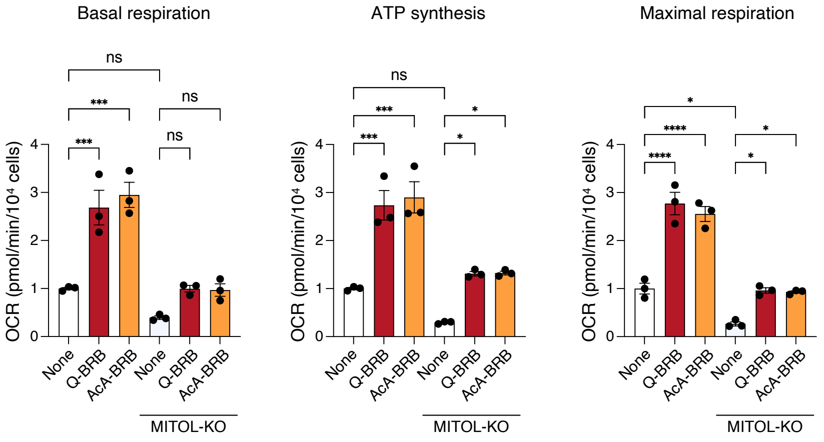

Supplementary Figure 10.
Evaluation of MITOL dependency in berberrubine-induced enhancement of mitochondrial oxygen consumption in C2C12 myoblasts.

C2C12 myoblasts (wild-type and MITOL-knockout) were treated with quinoid-type berberrubine (Q-BRB) or the berberrubine acetic acid adduct (AcA-BRB) (10 µM) for 48 h, and mitochondrial oxygen consumption rate (OCR) was measured (n = 3 per group). Basal respiration, ATP synthesis–linked respiration, and maximal respiration were calculated from the OCR data according to the method described in the Materials and Methods section. The None group indicates cells cultured without any additives, including solvents.
Statistical significance was assessed using one-way ANOVA followed by Tukey's HSD test. *p < 0.05; **p < 0.01; ***p < 0.001; ****p < 0.0001; ns, not significant.

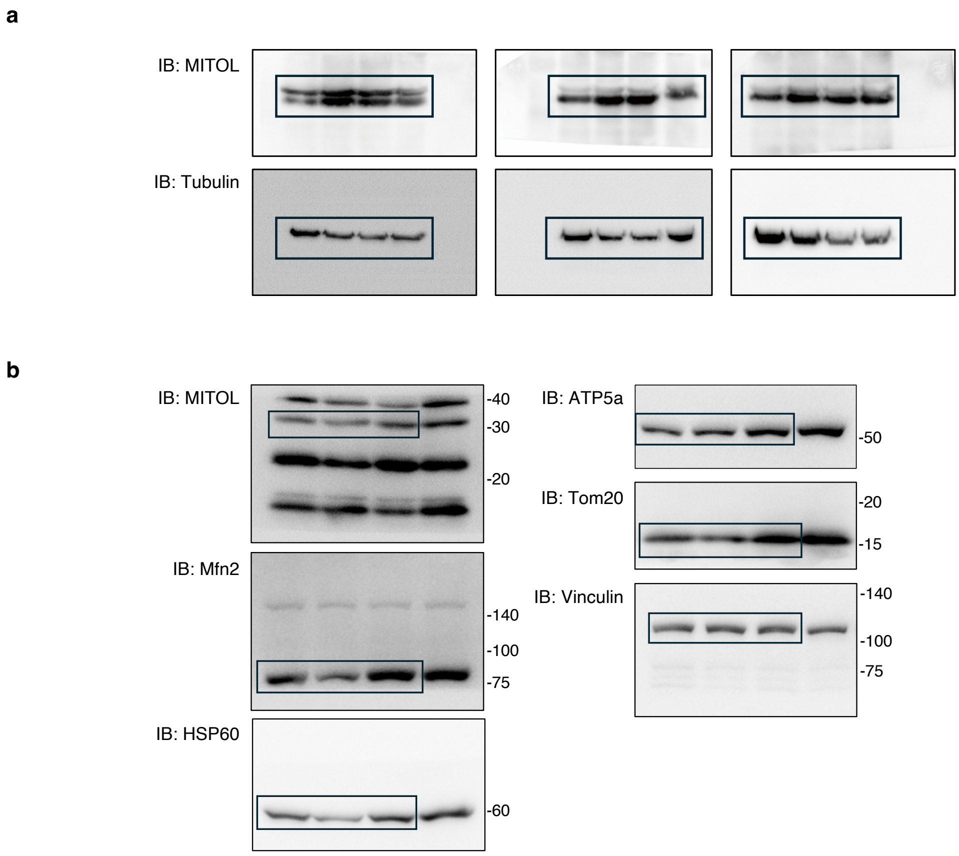

Supplementary Figure 11.
Unprocessed Western blot images related to (a) Figure 1c and (b) Figure 1e.

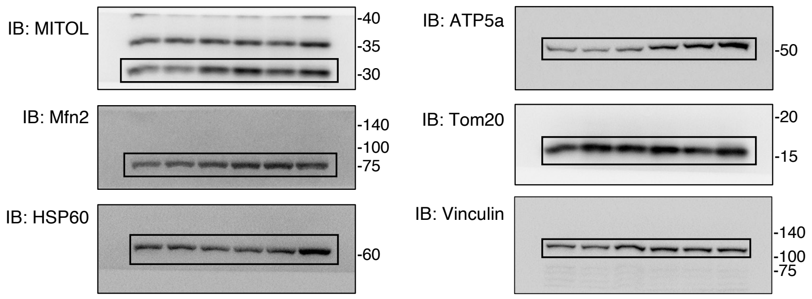

Supplementary Figure 12.
Unprocessed Western blot images related to Figure 3a.

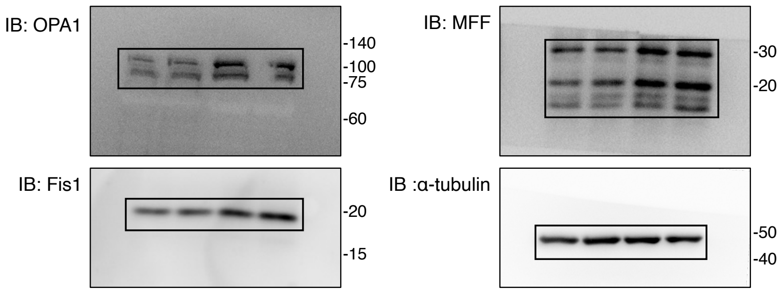

Supplementary Figure 13.

Unprocessed Western blot images related to Supplementary Figure 2a.

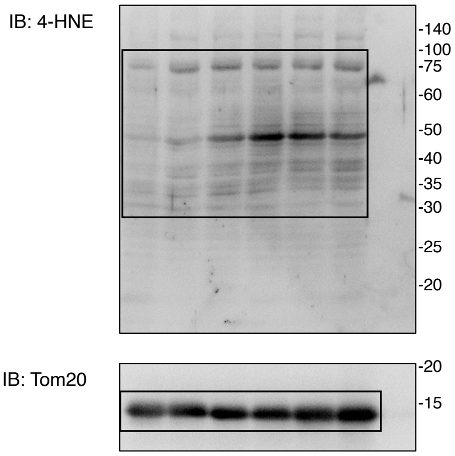

Supplementary Figure 14.
Unprocessed Western blot images related to Supplementary Figure 4a.

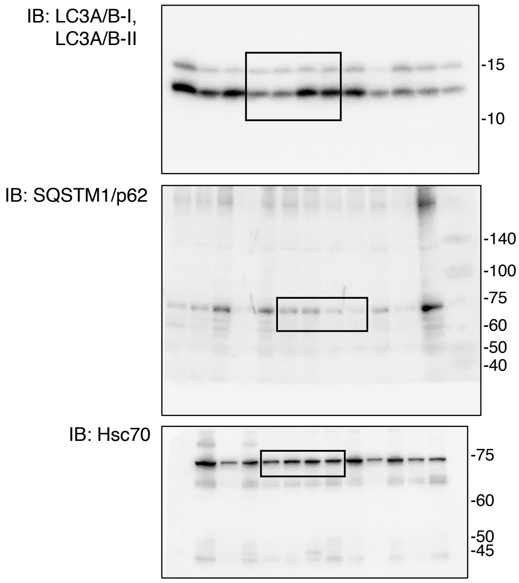

Supplementary Figure 15.
Unprocessed Western blot images related to Supplementary Figure 6.

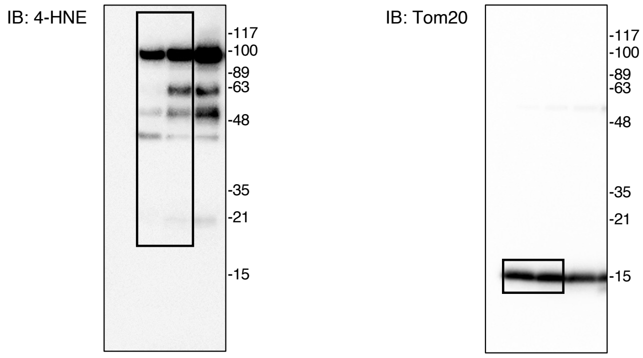

Supplementary Figure 16.
Unprocessed Western blot images related to Supplementary Figure 7.

Supplementary Table 1.
Primer pairs used for quantitative RT-PCR analysis in C2C12 cells.

qPCR targets	Primer directions	Sequences
Mitol / March5	Forward	5′-TTCACCAGGCTTGTCTCCA-3′
	Reverse	5′-GCATCACTGTCACTGCTCCA-3′
Tom20	Forward	5′-ATGGTGGGCCGGAACAGCGCCATCGCCGCGGGCGTG-3′
	Reverse	5′-TCATTCCACATCATCTTCAGCCAAGCTC-3′
Gapdh	Forward	5′-CCTGCACCACCAACTGCTTAGC-3′
	Reverse	5′-GCCAGTGAGCTTCCCGTTCAGC-3′

Supplementary Table 2.
Primer pairs used to quantify mitochondrial DNA content.

qPCR targets	Primer directions	Sequences
16S rRNA	Forward	5′-CCGCAAGGGAAAGATGAAAGAC-3′
	Reverse	5′-TCGTTTGGTTTCGGGGTTTC-3′
ND1	Forward	5′-CTAGCAGAAACAAACCGGGC-3′
	Reverse	5′-CCGGCTGCGTATTCTACGTT-3′
HK2 (Hexokinase 2)	Forward	5′-GCCAGCCTCTCCTGATTTTAGTGT-3′
	Reverse	5′-GGGAACACAAAAGACCTCTTCTGG-3′

Supplementary Table 3.
Primer pairs used for qRT-PCR analysis in mouse heart samples (1/2).

qPCR targets	Primer directions	Sequences
Rps18	Forward	5′-TTCTGGCCAACGGTCTAGACAAC-3′
	Reverse	5′-CCAGTGGTCTTGGTGTGCTGA-3′
Nppa	Forward	5′-GAGAGACGGCAGTGCTTCTAGGC-3′
	Reverse	5′-CGTGACACACCACAAGGGCTTAGG-3′
Nppb	Forward	5′-AGGCGAGACAAGGGAGAACA-3′
	Reverse	5′-GGAGATCCATGCCGCAGA-3′
Myh7	Forward	5′-CGGACCTTGGAAGACCAGAT-3′
	Reverse	5′-GACAGCTCCCCATTCTCTGT-3′
Col1a1	Forward	5′-GAGCGGAGAGTACTGGATCGA-3′
	Reverse	5′-CTGACCTGTCTCCATGTTGCA-3′
Ctgf	Forward	5′-CAAAGCAGCTGCAAATACCA-3′
	Reverse	5′-GGCCAAATGTGTCTTCCAGT-3′
Nrf1	Forward	5′-TTGCCCAAGTGAATTACTCTGCTG-3′
	Reverse	5′-TGCAGGACAGTCTGAGCCATC-3′
Nrf2	Forward	5′-TTGGCAGAGACATTCCCATTTGTA-3′
	Reverse	5′-AGTCATGGCTGCCTCCAGAGA-3′
Pgc1a	Forward	5′-CCGTAAATCTGCGGGATGATG-3′
	Reverse	5′-CAGTTTCGTTCGACCTGCGTAA-3′
Tfam	Forward	5′-TGAAGCTTGTAAATGAGGCTTGGA-3′
	Reverse	5′-CGGATCGTTTCACACTTCGAC-3′
Caren	Forward	5′-GCATCATGACTGCCTGGG-3′
	Reverse	5′-ATTTGTGTGTTCCTGGTGGG-3′
Mitol / March5	Forward	5′-CAGAGTGGCCTGTCCTCAGT-3′
	Reverse	5′-GGGCAAGCTTTTGAGATCAGT-3′
Mfn1	Forward	5′-CTACCTTATGACCGAAGGG-3′
	Reverse	5′-TGGAGGGCATGGGCCAGC-3′
Mfn2	Forward	5′-CCTGGGATCGATGTTACCAC-3′
	Reverse	5′-AACTGCTTCTCCGTCTGCAT-3′

Supplementary Table 3.
Primer pairs used for qRT-PCR analysis in mouse heart samples (2/2).

qPCR targets	Primer directions	Sequences
Opa1	Forward	5′-TAGGCGACTAGAGAAAAACG-3′
	Reverse	5′-CACAGTCTACTTCTCCTGGTG-3′
Fis1	Forward	5′-CCGGCTCAAGGAATATGAAA-3′
	Reverse	5′-ACAGCCAGTCCAATGAGTCC-3′
Drp1	Forward	5′-AACAGGCAACTGGAGAGGAA-3′
	Reverse	5′-GCAACTGGAACTGGCACAT-3′
Prkn	Forward	5′-AGGACACGTCGGTAGCTTTG-3′
	Reverse	5′-CACTGGAAGACCAGGACAGG-3′
Pink1	Forward	5′-GGAGAGTATGGAGCAGTTAC-3′
	Reverse	5′-TAGGGTGTGGGGCAAGCTGC-3′
Bnip3l	Forward	5′-CAACAACAACAACTGCGAGG-3′
	Reverse	5′-CCCATTTCCATTCTCATTGC-3′
Cu,Zn-SOD (SOD1)	Forward	5′-CAGCATGGGTTCCACGTCCA-3′
	Reverse	5′-CACATTGGCCACACCGTCCT-3′
Mn-SOD (SOD2)	Forward	5′-TCGCTTACAGATTGCTGCCT-3′
	Reverse	5′-CGTGCTCCCACACGTCAATC-3′
Nqo1	Forward	5′-GCCGAACACAAGAAGCTGGAAG-3′
	Reverse	5′-GGCAAATCCTGCTACGAGCACT-3′
Gclc	Forward	5′-ACACCTGGATGATGCCAACGAG-3′
	Reverse	5′-CCTCCATTGGTCGGAACTCTAC-3′
Gclm	Forward	5′-TCCTGCTGTGTGATGCCACCAG-3′
	Reverse	5′-GCTTCCTGGAAACTTGCCTCAG-3′
HO-1	Forward	5′-CAAGCCGAGAATGCTGAGTTCATG-3′
	Reverse	5′-GCAAGGGATGATTTCCTGCCAG-3′
